# Departure from Hardy Weinberg Equilibrium and Genotyping Error

**DOI:** 10.3389/fgene.2017.00167

**Published:** 2017-10-31

**Authors:** Bowang Chen, John W. Cole, Caspar Grond-Ginsbach

**Affiliations:** ^1^Department of Biology, Southern University of Science and Technology, Shenzhen, China; ^2^Department of Neurology, Baltimore VA Medical Center (VHA), University of Maryland School of Medicine, Baltimore, MD, United States; ^3^Department of Neurology, University of Heidelberg, Heidelberg, Germany

**Keywords:** association studies in genetics, Hardy Weinberg Equilibrium (HWE), heterozygosity, SNP quality control

## Abstract

**Objective:** Departure from Hardy Weinberg Equilibrium (HWE) may occur due to a variety of causes, including purifying selection, inbreeding, population substructure, copy number variation or genotyping error. We searched for specific characteristics of HWE-departure due to genotyping error.

**Methods:** Genotypes of a random set of genetic variants were obtained from the Exome Aggregation Consortium (ExAC) database. Variants with <80% successful genotypes or with minor allele frequency (MAF) <1% were excluded. HWE-departure (d-HWE) was considered significant at *p* < 10E-05 and classified as d-HWE with loss of heterozygosity (LoH d-HWE) or d-HWE with excess heterozygosity (gain of heterozygosity: GoH d-HWE). Missing genotypes, variant type (single nucleotide polymorphism (SNP) vs. insertion/deletion); MAF, standard deviation (SD) of MAF across populations (MAF-SD) and copy number variation were evaluated for association with HWE-departure.

**Results:** The study sample comprised 3,204 genotype distributions. HWE-departure was observed in 134 variants: LoH d-HWE in 41 (1.3%), GoH d-HWE in 93 (2.9%) variants. LoH d-HWE was more likely in variants located within deletion polymorphisms (*p* < 0.001) and in variants with higher MAF-SD (*p* = 0.0077). GoH d-HWE was associated with low genotyping rate, with variants of insertion/deletion type and with high MAF (all at *p* < 0.001). In a sub-sample of 2,196 variants with genotyping rate >98%, LoH d-HWE was found in 29 (1.3%) variants, but no GoH d-HWE was detected. The findings of the non-random distribution of HWE-violating SNPs along the chromosome, the association with common deletion polymorphisms and indel-variant type, and the finding of excess heterozygotes in genomic regions that are prone to cross-hybridization were confirmed in a large sample of short variants from the 1,000 Genomes Project.

**Conclusions:** We differentiated between two types of HWE-departure. GoH d-HWE was suggestive for genotyping error. LoH d-HWE, on the contrary, pointed to natural variabilities such as population substructure or common deletion polymorphisms.

## Introduction

Given that no genotyping method is 100% accurate and that genotype mistakes can lead to increased random error and bias in gene-disease associations (Gordon and Ott, 2001), methods have been developed to detect genotyping error. Tests of Hardy-Weinberg equilibrium (HWE) are widely used for a prompt check of genotype information (Tiret and Cambien, 1995; Xu et al., 2002; Hosking et al., 2004; Attia et al., 2010; Wang and Shete, 2012). This latter method is based on the assumption that in a large, randomly mating population, genotype frequencies should comply with HWE proportions. Deviation from these proportions can be caused by many factors, one of which is genotyping error. However, HWE-departure may also occur due to a variety of other causes, including purifying selection, copy number variation, inbreeding or population substructure (Lee et al., 2008; Wang and Shete, 2012; Graffelman et al., 2017). Such causes may vary over differing populations, and not accounting for such causes can result in inappropriate application of quality filtering strategies during GWAS (genome-wide association study) data preparation (Reed et al., 2015). In the current study we explored HWE-departure across a large multiethnic data set and associated HWE-departure with different SNP (single nucleotide polymorphism) characteristics, in order to find specific characteristics of HWE-departure due to genotyping error. Our findings are relevant to the GWAS methodology, including GWAS of neurological phenotypes, and highlight the importance of careful HWE-filtering.

## Methods

Using genetic variant data from the Exome Aggregation Consortium (ExAC) database (http://exac.broadinstitute.org/) we explored causes of HWE-departure in a diverse multiethnic human population (Lek et al., 2016). In brief, this data set includes 60,706 unrelated individuals sequenced as part of various disease-specific and population genetic studies, including several neurological disease phenotypes. Using ExAC data a random sample of genes was drawn by selecting a consecutive series of genes named “open reading frame” (C1orf1,2,3,4,…-100; C2orf1,2,3,4,…-100; etc.; C6orf1,2,3,4,…-50 until C22orf1,2,3,4,.-50). For each gene, we selected the variant with minor allele frequency (MAF) closest to 50% and with an identifier in the dbSNP database (https://www.ncbi.nlm.nih.gov/projects/SNP/). The sample (see Supplementary File) comprised 944 short sequence variants, genotyped across six super-populations: African (AFR, *n* = 5,302), Ad-mixed American (“Latinos”: AMR, *n* = 5,789), East Asian (EAS, *n* = 4,327), Finnish (FIN, *n* = 3,307), Non-Finnish European (NFE, *n* = 33,370) and South Asian (SAS, *n* = 8,256). From this initial study sample, 360 variants were excluded, because the site was covered in fewer than 80% of the individuals in ExAC, which may indicate a low-quality site. The final sample comprised 584 variants, typed in the six super-populations. In an additional analysis, all variants with genotyping rate <98% were excluded. Variants with population-specific MAF <0.001 were not analyzed in the respective super-population.

Departure from HWE was defined as *p*-HWE<10 E-05 and tested by χ^2^ test of goodness of fit between observed and expected genotypes according to the binominal distribution: m^2*^AA; 2m^*^(1 − m)^*^AB; (1 − m)^2*^BB. For each super-population we calculated the ratio between the observed and expected (O/E) number of heterozygous carriers assuming HWE-equilibrium. HWE-departure (d-HWE) associated with reduction of heterozygosity frequency (“loss of heterozygosity”: LoH d-HWE) was analyzed separately from HWE-departure associated with excess of heterozygote carriers (“gain of heterozygosity”: GoH d-HWE). Variants with genotypes in HWE were considered as “control” SNPs and compared with both types of HWE-violating variants by statistical tests, as specified in the legends of Table 1 [*p*-values for comparison of d-HWE groups with variants without HWE-departure by Mann-Whitney test (Missing genotypes, MAF, MAF-SD) or χ^2^ test (others)]. For replication and extension of our findings, we explored HWE-departure in a large sample short variants of chromosome 17, and common deletion on chromosome 3, using data from the 1000 Genomes Project (1000 Genomes Project Consortium et al., 2015).

**Table 1:** Causes of HWE departure associated with reduced or excess heterozygotes.

		**No d-HWE (3070)**	**LoH d-HWE (41)**	***p***	**GoH d-HWE (93)**	***p***
Missing genotypes (med, IQ range)		0.56 (2.62)	0.26 (2.77)	0.486	13.58 (8.92)	<0.001
Insertion/deletion type (*n*, %)		133 (4.1)	0	0.415	29 (19.7)	<0.001
MAF (med, IQ range)		0.327 (0.237)	0.271 (0.205)	0.252	0.407 (0.134)	<0.001
MAF-SD between populations		10.57 (6.76)	13.26 (7.88)	0.0077	12.34 (6.14)	0.122
CNV-loss (*n*, %)		9 (0.3)	4 (9.8)	<0.001	0	1.00
CNV-gain (*n*, %)		11 (0.3)	0	1.00	0	1.00
	AFR	533	3		7	
	AMR	509	19		15	
Heterogeneity across populations	EAS	491		2	10	
	FIN	528	2	<0.001	7	<0.001
	NFE	493	5		45	
	SAS	516	10		9	

*HWE-departure (d-HWE) was defined as p-HWE < 10E-5. LoH d-HWE: loss of heterozygosity associated HWE-departure. GoH d-HWE: gain of heterozygosity associated HWE-departure. AFR, African; AMR, Latino; EAS, East Asian; FIN, Finnish; NFE, Non-Finnish European; SAS, South Asian; MAF, minor allele frequency; SD, standard deviation; IQ range, interquartile range; CNV, copy number variation). P-values for comparison of d-HWE groups with variants without HWE-departure by Mann-Whitney test (Missing genotypes, MAF, MAF-SD) or χ^2^ test (others)*.

Moreover, for each variant the following five variables were defined:

1. Percentage of missing genotypes: the total number of non-genotyped subjects in the ExAC population, divided by 60,706 (the total number of individuals in the ExAC);
2. Variant type: single nucleotide polymorphism (SNP) versus insertion/deletion polymorphism (indel);
3. Minor allele frequency (MAF) for each variant, specified for the total ExAC sample each and for each super-population;
4. Standard deviation (SD) of MAF among the super-populations for each variant (MAF-SD). This item was assessed as a surrogate marker for population substructure, as we hypothesized that SNPs with strongly varying MAF-values across super-populations might be more likely to have varying MAF-values within a sub-population as well;
5. Copy number variation (“gain”-duplication or “loss”-deletion) with MAF > 0.1%.

## Results

The final study sample comprised 3,204 genotype distributions (584 variants, typed across six super-populations), downloaded from the ExAC database. Significant HWE-departure (at *p* < 10e-05 level) was observed in 134 (4.2%) variants. HWE-departure was associated with an excess of heterozygotes (GoH d-HWE) in 93 variants, and with a loss of heterozygosity (LoH d-HWE) in 41 variants. Predictors of HWE-departure were identified by comparing both categories of HWE-violating variants with non-HWE-violating variants as demonstrated in Table 1. LoH d-HWE was significantly associated with localization within a deletion polymorphism (CNV-loss: *p* < 0.001) and with increased MAF-SD (*p* = 0.008). Lower genotyping rate (missing genotypes) and insertion/deletion polymorphisms, on the other hand, were associated with GoH d-HWE (both at *p* < 0.001). Significant differences in heterogeneity across the super-populations was found in both types of HWE-departure.

Table 2 presents some typical examples of HWE-violating variants. Variant rs7551421, a SNP in C1orf62 (encoding the AKNA domain 1 containing *AKNAD1*) is located within the common deletion polymorphism esv3587138. In the overall ExAC population (*n* = 60,706 subjects), this variant had MAF = 0.45 and MAF-SD = 0.12 and was successfully genotyped in 60,584 (99.80%) subjects. The deletion allele removes a single exon from the C1orf62 gene. In the 1000 Genomes Project database, this deletion was not found in the Finnish population and was virtually absent from Africans, but occurred at low frequency in the other populations (http://www.ensembl.org/Homo_sapiens/StructuralVariation/Explore?r=1:108823471-108830221;sv=esv3587138;svf=53210844;vdb=variation).

**Table 2:** Examples of HWE-departure in three ExAC variants.

**Population**	**Obs:AA**	**Obs:AB**	**Obs:BB**	**Exp:AA**	**Exp:AB**	**Exp:BB**	**MAF**	***p*-HWE**	**Ratio O/E**	**d-HWE type**
**C1orf62 (rs7551421): variant located within deletion polymorphism**										
African	691	2,455	2,051	708	2,421	2,068	0.37	0.59	1.01	
East Asian	2,392	1,463	429	2,277	1,692	314	0.73	8.4 E-18	0.86	LoH
European (Finnish)	864	1,630	812	853	1,653	801	0.51	0.73	0.99	
European (Non-Finnish)	5,564	15,393	12,372	5,276	15,969	12,084	0.40	3.8 E-10	0.96	LoH
Latino	1,658	2,593	1,523	1,512	2,885	1,377	0.51	1.3 E-13	0.90	LoH
South Asian	2,149	3,760	2,333	1,970	4,119	2,154	0.49	2.6 E-14	0.91	LoH
**C11orf10 (rs509360): variant with strongly different allele frequency across populations**										
African	3,552	1,468	173	3,537	1,497	158	0.83	0.37	0.98	
East Asian	968	1,914	1,434	859	2,133	1,325	0.45	1.4 E−10	0.90	LoH
European (Finnish)	416	1,503	1,380	413	1,509	1,377	0.35	0.98	1.00	
European (Non-Finnish)	3,423	14,518	15,378	3,425	14,515	15,380	0.32	1.00	1.00	
Latino	195	1,396	4,190	138	1,510	4,133	0.15	6.8 E−08	0.92	LoH
South Asian	4,205	3,292	749	4,152	3,399	696	0.71	0.02	0.97	
**C1orf31 (rs58896934): variant of deletion-insertion type**										
African	110	1,620	2,198	215	1,409	2,303	0.23	7,6 E−20	1.15	GoH
East Asian	829	2,124	622	1,000	1,782	793	0.53	2,0 E−29	1.19	GoH
European (Finnish)	917	1,632	416	1,013	1,440	512	0.58	3,8 E−12	1.13	GoH
European (Non-Finnish)	5,685	16,989	4,949	7,279	13,802	6,543	0.51	0	1.23	GoH
Latino	531	2,895	1,412	809	2,339	1,690	0.41	3.8 E−60	1.24	GoH
South Asian	1,943	4,079	941	2,278	3,409	1,276	0.57	4.8 E−59	1.20	GoH

*Obs, Observed genotypes; Exp, expected genotypes under hypothesis of HWE; Ratio O/E, ratio of observed and expected number of heterozygote carriers; p-HWE, p-value of χ^2^ test for HWE-departure; MAF refers to minor allele frequency as seen in the entire ExAC sample; in some superpopulations this “minor allele” may be the common allele*.

Consistent with these findings there was no deviation from HWE in the ExAC African or Finnish populations. For each of the other ExAC super-populations the allele frequency of the deletion polymorphism and observed loss of heterozygosity (ratio O/E) were inversely correlated.

SNP rs509360 in C11orf10 illustrates population stratification and was successfully genotyped in 60,606 (99.83%) subjects. It had MAF = 0.41 and MAF-SD = 0.23. HWE-violation was observed in the East Asian (EAS) and Latino (AMR) super-populations. Indeed, analysis of sub-populations from the 1000 Genomes Project database revealed significant substructure (stratification) within these EAS subpopulations, explaining HWE-departure (http://www.ensembl.org/Homo_sapiens/Variation/Population?db=core;r=11:61780587-61781587;v=rs509360;vdb=variation;vf=344594). Within the AMR superpopulation, the following allele frequencies were found for rs509360: Colombians from Medellin, Colombia (CLM): 0.24, Mexican Ancestry from Los Angeles USA (MXL): 0.14; Peruvians from Lima, Peru (PEL): 0.12, Puerto Ricans from Puerto Rico (PUR): 0.28, and within the East Asian superpopulation: Chinese Dai in Xishuangbanna, China (CDX): 0.21; Han Chinese in Bejing, China (CHB): 0,65; Southern Han Chinese (CHS): 0.40; Japanese in Tokyo, Japan (JPT): 0.67 and Kinh in Ho Chi Minh City, Vietnam (KHV): 0.17.

SNP rs58896934 in C1orf31 illustrates HWE-violation due to genotyping error. This insertion/deletion variant (MAF = 0.50, MAF-SD = 0.12) was successfully typed in 50,280 (82.83%) subjects. In all populations, a significant excess (gain) of heterozygosity was observed.

To minimalize the impact of genotyping error, a subsequent investigation was performed in which only variants with genotyping rates higher than 98% were selected. Among the analyzed 2,196 genotype distributions, HWE-departure associated with excess heterozygotes (GoH d-HWE) was not observed. However, 29 SNPs in this sample of high quality genotypes showed HWE-departure of the LoH subtype, among them 15 variants in the AMR (Latino) and 9 variants in the South Asian super-populations.

For replication and extension of our findings, we first explored HWE-departure in a large sample short variants of chromosome 17 in 503 the European individuals from the 1000 Genomes Project. An unfiltered set of 2,317,399 short variants of chromosome 17 was downloaded and 260,671 variants with MAF > 0.01 were selected for further analysis. Significant HWE-departure (at *p* < 10E-05) was observed in 3,942 (1.5%) variants. Among the 260,671 analyzed variants, 31,650 were indels and 229,021 were true SNPs. Significant HWE-departure was found in 1,281 (4.0%) indels and in 2,661 (1.2%) true SNPs, revealing that the analyzed indels were more likely (χ^2^ test *p* < 0.001) to violate HWE (compared to the true SNPs). Interestingly, all short variants from the 1000 Genomes Project were found to be completely genotyped (i.e., in all 503 subjects), even those variants that were located in common deletion polymorphisms and that were likely to have been totally absent (homozygous deletion) in some individuals. Because no missing genotypes were reported in this dataset, we could not confirm the association between incomplete genotyping and HWE-violation in the 1000 Genomes Project dataset.

**Figure 1:**
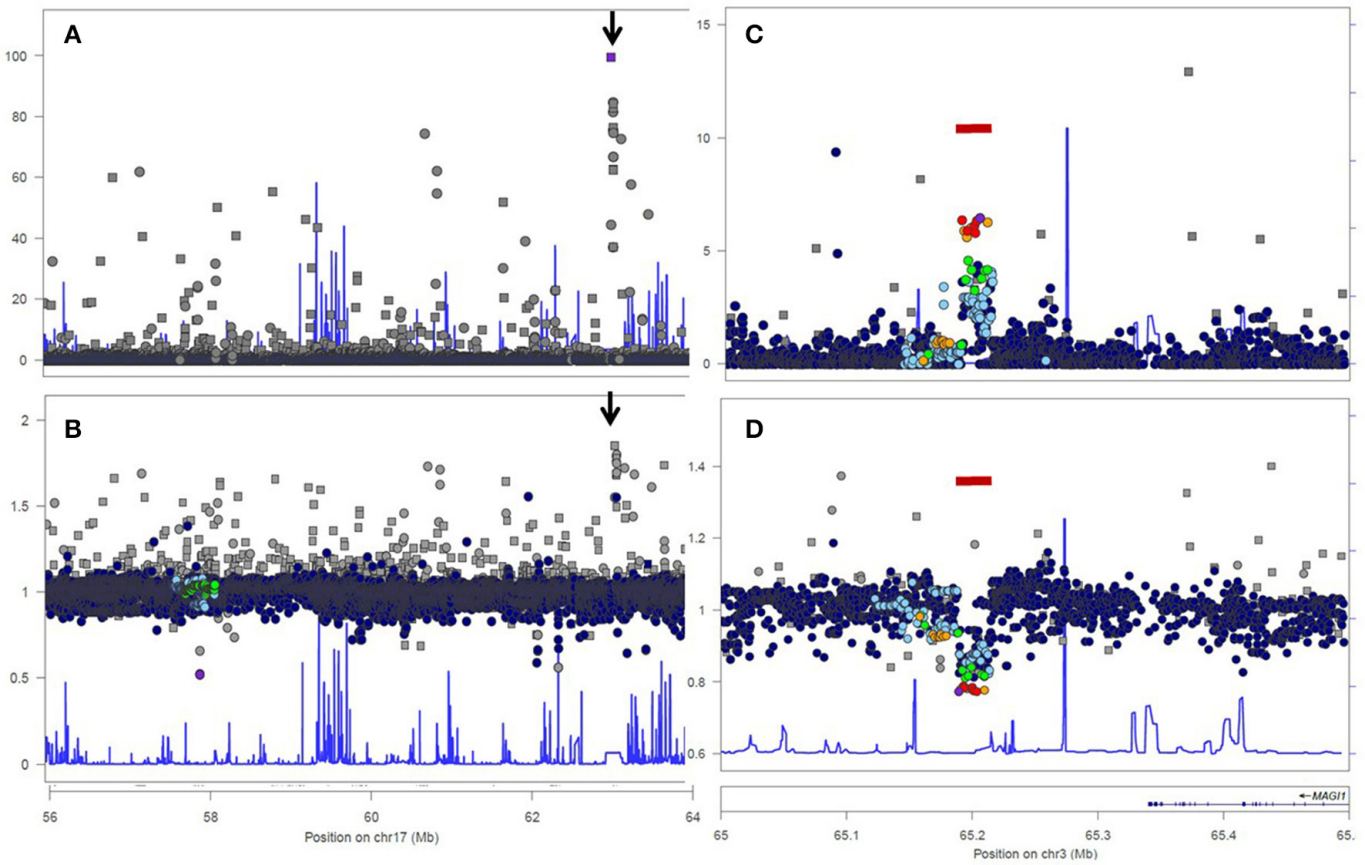
Analysis of genotyped short variations in genomic region 17:56,000,000–64,000,000 **(A,B)** and genomic region 3:65,000,000–65,500,000 **(C,D)** from the 1000 Genomes Project, European Population. Upper figures **(A,C)** show log-transformed *p*-values for departures from HWE. Lower figures **(B,D)** show ratios of observed and expected (i.e., under Hardy-Weinberg Equilibrium) frequencies of heterozygous genotypes. Dots indicate true SNPs, rectangles symbolize variants of indel type. The bar (seen in **C,D**) indicates common deletion variant esv2657253, (http://www.ensembl.org/Homo_sapiens/StructuralVariation/Explore?r=3:65202694--65229573;sv,esv2657253;svf,3513219;vdb,variation). The arrow (seen in **A,B**) points to a region with of a degenerate repeat structure within the 5' end of the LRRC37A2 with strong homology with sequences of the LRRC37A3 gene, resulting in genotyping error.

Analysis 1000 Genome data for a 20Mb genomic region of chromosome 17 confirmed the highly non-random distribution of HWE-violating SNPs across the genome. Most—but not all—SNPs with strong HWE-departure showed excess of heterozygotes (Figure 1B), but the median O/E ratio was slighly below 1.00, due to a low degree of population structure, inbreeding, and limited effective population size. A peak of 11 SNPs with strong HWE-departure was observed around position 17:62.9 (arrows in Figures 1A,B). All 11 SNPs showed an unexpectedly high frequency of heterozygous genotypes (data no shown).

These 11 short genetic variants with highly significant departures from HWE were mapped on a 2.4 Kb fragment, which was analyzed by a BLAST-search to find sequence homology elsewhere in the human genome. a highly homologous sequence was found on chromosome 17, within the 5′ end of the LRRC37A2 gene. Interestingly, the sequences of the LRRC37A2 and the LRRC37A3 copies differed for the identified 11 variants. As a consequence, each individual is necessarily heterozygous for these SNPs. Some of these variants (for instance: indel rs71828933 and SNP rs11650755) were classified as “suspect” in the 1000 Genomes Project database.

Next, we worked to replicate and further demonstrate the non-random distribution of HWE-violating SNPs along the genome due to local loss of heterozygosis in common deletions. This can be easily observed in a local Manhattan plots of HWE-violation (see Figures 1C,D) of chromosome 3:65,000,000–65,500,000. We selected 1000 Genomes Project SNPs from this region for this analysis, because deletion polymorphism esv2657253 is very common and expected to cause significant HWE-departure even in a sample of limited size (*n* = 503). Indeed, all SNPs within the deletion polymorphism showed HWE-departure (Figure 1C) and loss of heterozygosity (Figure 1D) in the European population.

## Discussion

In the current analysis of a random large sample of genetic variants in different human populations, those with lower genotyping rates (< 98%) and insertion/deletion polymorphisms were more likely to violate HWE than genuine SNPs with genotyping rates >98%. This finding is in line with the well-known observation that genotyping error is an important cause of HWE-departure (Tiret and Cambien, 1995; Xu et al., 2002; Hosking et al., 2004; Attia et al., 2010). To increase the specificity of HWE filtering, we made the distinction between HWE-departure associated with excess (gain) of heterozygotes (GoH d-HWE) and HWE-departure associated with loss of heterozygotes (LoH d-HWE). Genotyping error appeared to be specifically associated with GoH d-HWE, but not with LoH d-HWE. This finding may be related to allelic dropouts. For example, if one assumes 2 alleles (A, a) both with an allele frequency of 0.5, the genotype distribution would be 25% AA, 25% aa, and 50% Aa. As such, it would be equally likely that an allelic genotyping error could create a “false” homozygote or a heterozygote. However, when measuring the error rate per locus, allelic dropouts are less likely to be detected at homozygous loci (a heterozygous locus affected by allelic dropout and a true homozygous locus will both appear as a single band or peak), and therefore heterozygotes would be more likely to be detected, hence leading to the gain of heterozygosity. LoH d-HWE, on the contrary, was associated with real existing biological phenomena including deletion polymorphisms and population substructure. This key observation of our study suggests that the specificity of HWE-testing to detect genotyping error is increased by differentiating between HWE-departure associated with excess or lack of heterozygous carriers. In our sample a loss of heterozygosity found to be associated with deletion polymorphisms. Within deletions, hemizygosity occurs and heterozygosity is excluded, which leads to an overall reduction of heterozygosity in genomic regions with deletion polymorphisms. Notably, inbreeding may also result in a loss of heterozygosity. Long genomic runs of homozygosity are typically found in the genomes of children from consanguineous parents (McQuillan et al., 2008). Populations with frequent consanguineous marriages may therefore demonstrate a noticeable reduction of the overall heterozygosity frequency. Inbreeding was found in all 26 populations of the 1000 Genomes Project, with particularly high levels of inbreeding in the SAS and AMR superpopulations (Gazal et al., 2015). Our finding of HWE-departure in these two populations even after selection of high quality genotyping data (only variants with successful genotypes in >98% of the sample were included) may be related to the higher levels of inbreeding in these populations. However, many other causes of population substructure are known to occur, including: socio-economic, geographic, religious, political, and ethnic affiliations that may significantly restrict partner selection (Lupski et al., 2011). In fact, random mating, albeit the central assumption for HWE, does not apply to human populations, as clearly appreciated by Weinberg in his initial publication (Weinberg, 1908).

The skewness of the distribution of O/E ratios was an unexpected observation of our study, and in agreement with recent observations by others (Graffelman et al., 2017). The example of the 11 short “suspect” variants in the LRRC37A2 may illustrate one of the causes for this assymetry: in fact these putative “suspect” variants are to be removed from the database: they are no variants between genomes, but between degenerate sequence copies within one and the same genome. We cannot exclude that other putative HWE-violating SNPs with excess heterozygotes are in fact misclassified unspecific cross-hybridizations.

Strengths of our study include the diverse populations evaluated, the high-quality data as attained from the ExAC database (Lek et al., 2016) and the validation of our findings in a large set of short variants from the 1000 Genomes Project (1000 Genomes Project Consortium et al., 2015). Study weaknesses might include the limited number of variants included and whether these genes are an accurate representation of the entire genomic architecture, this across the differing super-populations. Further, the current study does not evaluate HWE-departure in extremely rare variants, although the genotype distributions of extremely rare variants are particularly likely to violate HWE.

## Conclusion

Two categories of HWE-departure were studied in the ExAC populations. HWE-departure associated with loss of heterozygosity (LoH d-HWE) may be explained by natural or biological causes, including genomic deletions, population stratification and inbreeding. On the other hand, HWE-departure associated with gain of heterozygosity (GoH d-HWE), may indicate genotype error. For the detection of genotyping error, testing of HWE should be refined and combined with the analysis of observed heterozygosity frequencies.

## Author contributions

BC and CG attained the data, performed the analyses, critically interpreted the data and prepared the manuscript. JC critically interpreted the data and prepared the manuscript.

### Conflict of interest statement

The authors declare that the research was conducted in the absence of any commercial or financial relationships that could be construed as a potential conflict of interest.
